# Feasibility and psychometric analysis of graduate satisfaction survey of medical students graduating from the Royal College of Surgeons in Ireland-Medical University of Bahrain (RCSI Bahrain)

**DOI:** 10.1186/s40064-016-1957-3

**Published:** 2016-03-10

**Authors:** Kathryn Strachan, Ahmed Al Ansari

**Affiliations:** Royal College of Surgeon of Ireland in Bahrain, Building No 2441, Road 2835, Busaiteen, 228 Kingdom of Bahrain; Department of General Surgery, Bahrain Defense Force Hospital, Off Waly Alahed Avenue, P. O. Box - 28743, West Riffa, Kingdom of Bahrain; Arabian Gulf University, Building 293, Manama, 329 Kingdom of Bahrain

**Keywords:** Graduate survey, Validity, Reliability

## Abstract

To assess the satisfaction levels of graduates of the Royal College of Surgeons in Ireland University of Bahrain (RCSI Bahrain). The graduate survey was administered to four groups of graduates of the RCSI Bahrain who graduated between the years 2010 and 2014. The graduate survey assessed five major domains and comprised 41 items. The RCSI Bahrain opened its doors in 2004, with the first class graduating in 2010. The graduate cohorts used in this study were working in various countries at the time of survey completion. Out of 599 graduates, 153 responded to the graduate survey. The total mean response rate of the graduate survey was 26 %, including 102 females, 44 males, and 7 students who did not indicate their gender. 49 students graduated in 2012, and 53 students graduated in 2013. Of these graduates, 83 were working in Bahrain at the time of survey administration, 11 in the USA, 4 in Malta, and 3 in the UK; the total number of countries where graduates were working was 14. Reliability analysis found high internal consistency for the instrument (with a Cronbach’s α of 0.97). The whole instrument was found to be suitable for factor analysis (KMO = 0.853; Bartlett test significant, p < 0.00). Factor analysis showed that the data on the questionnaire decomposed into five factors, which accounted for 72.3 % of the total variance: future performance, career development, skills development, graduate as collaborator, and communication skills. The survey results found that graduates of the RCSI Bahrain program who responded to this questionnaire are generally satisfied with their experience at the university, feel well prepared to join the field and feel ready to compete with graduates of competing universities. Furthermore, the graduate survey was found to be a reliable instrument and we provided some evidence to support the construct validity of the instrument.

## Background

Upon graduation from medical school, it is important for universities to monitor student satisfaction, to build on constructive feedback and to further improve the quality of education offered. The Graduation Questionnaire was first established by the Association of American Medical Colleges (AAMC) in 1978, and medical schools receive the results of both their institution as well as other medical programs nationwide for comparison (Association of American Medical Colleges 2012). The Medical School Graduation Questionnaire administered by the AAMC produces an annual report that analyzes a variety of aspects of graduate experiences. The questionnaire covers medical education topics, clinical experiences, preparedness for residency, financing of education, and overall satisfaction, amongst others (Mavis et al. 2014).

Past research has established that more recent cohorts of medical school graduates tend to rate higher satisfaction than previous ones (Lambert et al. 2013). This is likely due to the notion that medical schools are continuously improving their respective programs based on feedback from their graduates. Previous studies have also indicated that new physicians often find difficulty in the areas of time management, paperwork, inpatient ward work and working on-calls; however these are areas that the authors argue are best learned through experience rather than the type of training learned in a scholarly setting (Illing et al. 2013). As such, this questionnaire is useful both as an evaluation method as well as a quality improvement process.

Such surveys are an important tool for developing medical education programs that are catered to the specific needs as seen from a student’s perspective. These questionnaires may then be used to improve rates of graduate preparedness for practicing medicine at the residency level. Similar questionnaires have been used in a variety of countries to change the medical curriculum. For instance, a study conducted in Poland found that students who shadowed surgeons in the operating room, observing the procedures rather than participating in them, found this learning method to be inadequate to prepare them for performing surgery themselves (Lambert et al. 2013). As such, the curriculum evolved to include hands-on training using surgical simulators (Illing et al. 2013). The purpose of this study therefore was to assess the satisfaction and preparation levels of graduates of the RCSI Bahrain.

The RCSI Bahrain opened its doors in 2004, with the first class graduating in 2010. The RCSI was first established in Dublin, Ireland in 1784. Medical interns graduating from RCSI Bahrain have established a 100 % pass rate for the Bahrain National Health Regulatory Authority licensing exams thus far. For the most part, staff and faculty are primarily from Bahrain and Ireland, respectively.

In 2013, the medical school was invited to join the Global Education in Medicine Exchange (GEMx) affiliated to the Educational Commission for Foreign Medical Graduate (ECFMG) and the Global Health Learning Opportunities Collaborative (GHLO), affiliated to the Association of American Medical Colleges (AAMC). Both initiatives provide a network of collaborating institutions around the world, facilitating global mobility for our medical students pursuing international clinical or research electives. In 2014 there were more than 1278 students from 36 different countries and a total of 138 fulltime staff.

The aim of this study therefore was to assess the satisfaction and self-reported preparation levels of graduates of the Royal College of Surgeons of Ireland University of Bahrain (RCSI Bahrain).

## Methods

### Study settings and participants

The study included all of the students who graduated from the RCSI-Bahrain in the Kingdom of Bahrain. The participants included 102 female and 44 male graduates, to a total of 146 participants. The RCSI Bahrain opened its doors in 2004, with the first class graduating in 2010. In 2014 there were more than 1278 students from 36 different countries.

The graduate survey was administered via email to a total of 599 students; these students comprise four cohorts of graduates of the RCSI Bahrain who graduated between the years of 2010–2014. The graduates used in this study were working in various countries at the time of survey completion. The first e-mail was sent to the graduates by December 2014. This was followed by another two e-mails within a three-week interval. Graduates were contacted through their RCSI e-mail that was assigned to them by the university during the study. The name of the graduates was not mandatory for completing the survey. Confidentiality of the participants was preserved.

### Instrument

The graduate survey is a tool that was developed by an expert team from the RCSI in Dublin. Face and content validity was established by expert opinion and a table of specification. We addressed face and content validity by sending the instrument to an expert in the field to review the content and format of the instrument and judge whether or not it is appropriate. The questions of the survey were assessed against the table of specification, which was constructed by the authors.

The survey comprised of 41 items and assessed five major domains ranged on a 5-point likert scale. The items on the instrument had a 5-point response scale in the form of, 1 = strongly disagree; 2 = disagree; 3 = neither agree nor Disagree; 4 = agree; 5 = strongly agree” with an option of “unable to assess” UA. After the committee had developed the questionnaire, they were sent to group of physicians whose work fit the profile for episodic care, for feedback. The questionnaire was modified following that feedback. The items in the questionnaire are presented in Table 1.

**Table 1:** Descriptive statistics, item analysis and factor analysis

Question	Mean	SD	Future performance	Career development	Skills development	Graduates as collaborator	Communication skills
Q1 My programme prepared me for lifelong learning	4.10	0.87	0.540				
Q2 I am able to use reflection to enhance my professional development	4.10	0.73				0.611	
Q3 I am able to manage my time effectively	4.15	0.78			0.493		
Q4 I understand the role of the health care professionals in the team I work with	4.31	0.78			0.473		
Q5 I have achieved my professional goals at this stage in my career	3.50	1.21		0.704			
Q6 My programme of study enhanced my chances of employment	3.70	1.18			0.537		
Q7 I feel prepared to pursue postgraduate study	4.03	0.97			0.642		
Q8 My programme of study was relevant to the type of employment I pursued	4.11	0.81				0.435	
Q9 My chosen career has met my expectations	3.75	1.20		0.615			
Q10 Career development was an integral part of my programme at RCSI Bahrain	3.25	1.36		0.611			
Q11 The career guidance offered at RCSI Bahrain helped me gain the right type of experiences for my chosen career path	3.10	1.41		0.720			
Q12 I have engaged in continuous professional development since my graduation	3.99	0.88				0.490	
Q13 My programme helped me develop my leadership skills	4.00	0.97	0.615				
Q14 1My subject knowledge and understanding was sufficient for employment	4.13	0.82			0.795		
Q15 The curriculum was up to date and relevant to practice	4.16	0.88			0.653		
Q16 I am familiar with the RCSI Bahrain graduate profile for my profession	3.63	1.14		0.623			
Q17 My programme prepared me for research, scholarship and enquiry	3.53	1.32		0.818			
Q18 I am able to incorporate relevant research findings into healthcare practice	4.01	0.85		0.554			
Q19 The VLE was essential in supporting the development of my knowledge and understanding	4.23	0.82	0.581				
Q20 The time I had to practice subject specific skills was sufficient	3.69	0.91			0.608		
Q21 Professionalism was an important topic area in my programme of study	4.30	0.80	0.589				
Q22 My problem solving skills were developed during my programme	4.12	0.82	0.630				
Q23 My programme helped me develop my decision making skills	4.05	0.91	0.726				
Q24 The clinical practice in my programme was sufficient for me to enter my profession	3.87	1.11			0.656		
Q25 I am able to contribute to effective multidisciplinary teamwork	4.36	0.65				0.656	
Q26 I can establish and maintain constructive working relationships with colleagues	4.42	0.67				0.668	
Q27 My clinical experience enabled me to make career choices	3.95	1.02		0.560			
Q28 My understanding of cultural diversity in health care was enhanced during my time at RCSI Bahrain	4.23	0.84	0.528				
Q29 I understand health and social care policies on National and International level	3.87	0.93	0.501				
Q30 I am able to speak more than one language	4.31	0.78				0.754	
Q31 I was able to develop my language skills while at RCSI Bahrain	4.10	1.05					*0.633*
Q32 RCSI Bahrain helped me to develop my skills to communicate effectively with patients and staff	4.33	0.84			0.658		
Q33 RCSI Bahrain helped me to develop good oral and written communication skills	4.27	0.79					*0.560*
Q34 RCSI Bahrain helped me to develop skills to be adaptable and flexible in my approach to work	4.03	0.97	0.630				
Q35 RCSI Bahrain helped me to develop information technology skills that enable me to use in my practice	3.87	1.10					*0.636*
Q36 RCSI Bahrain helped me to develop my organizational skills	4.02	0.92	0.739				
Q37 I am self motivated	4.41	0.73				0.716	
Q38 RCSI Bahrain encouraged independence in teaching and learning	4.14	0.90		0.554			
Q39 RCSI Bahrain supported me in developing confidence in practice	4.16	0.93			0.549		
Q40 My interpersonal skills were developed at RCSI Bahrain	4.07	0.91	0.720				
Q41 I was encouraged to be part of wider community engagement whilst at RCSI Bahrain	3.79	1.08	0.567				

### Statistical analysis

Data analysis was conducted with SPSS version 20.0. Each research question underwent a number of statistical analyses. Feasibility of the questionnaire was analyzed via response rates and the average time required to complete the form. For each question on the surveys, the percentage of individuals who responded “unable to-assess,” along with the mean and standard deviation, was computed to determine the viability of the items and the score profiles. Items in which more than 20 % of responders selected “unable-to-assess” may be in need of revision or deletion according to past findings (Violato et al. 2008).

We used exploratory factor analysis to determine which items on each survey belonged together (i.e., becoming a factor or scale). In this study, using individual-physician data as the unit of analysis for the survey, the items were inter-correlated using Pearson product moment correlations. The correlation matrix was then decomposed into principal components, and these were subsequently rotated to the normalized varimax criterion. Items were considered to be part of a factor if their primary loading was on that factor. The number of factors to be extracted was based on the Kaiser Rule (i.e., eigenvalues >1.0) (Violato and Saberton 2006).

The factors or scales established through exploratory factor analysis were used to establish the key domains (e.g., Communication skills) for improvement, whereas the items within each factor provided more precise information about specific behaviors (e.g., RCSI Bahrain helped me to develop good oral and written communication skills). This analysis made it possible to determine whether the instrument items were aligned into the appropriate constructs (factors) as intended. Each item was assigned to the factor on which they loaded with at least a loading factor of 0.40. If an item loaded in more than one factor (cross-loading) the item was assigned to where it loaded the highest factor (Violato and Saberton 2006). The results of the factor analysis are presented in Table 1.

Instrument reliability (stability) was assessed. Internal consistency reliability was examined by calculating the Cronbach’s coefficient for the total scales and for each factor. This calculation provided an assessment of the overall internal consistency for each instrument as well as for each factor within the instruments (Lockyer et al. 2009).

### Ethical approval

The research was approved by the research ethics committee at the RCSI-MUB. Written consent was obtained from the graduates. This study was conducted from March 2014 to July 2014.

## Results

Out of 599 graduates, 153 responded to the graduate survey. The response rate of the graduate survey was 26 %, including 67 % females (n: 102), 29 % males (n: 44), and 5 % (n: 7) students who did not indicate their gender. These responses corresponding to the proportion of females in the university since female are presenting 60 % of the total number of graduates. There was a steady increase in response rates with each graduating class. There were a disproportionately higher number of respondents from more recent graduating years, representative of larger cohort sizes. This was also the case due to the ease of access to more recent graduates relative to alumni who graduated a few years prior to this study.

The majority of respondents (83) were working in Bahrain at the time of survey administration (54 %), followed by 11 students in the USA (7 %), 10 students in Canada (7 %), 7 in the United Arab Emirates (5 %), 6 in Saudi Arabia (4 %), 4 in Malta (3 %), and 3 in the UK (2 %); the total number of countries where graduates were working was 14. Other countries represented include Sweden, Qatar, Nigeria, Mali, Kuwait, Germany, and Afghanistan. 17 respondents did not answer this question, leading to 11 % of unaccounted for results.

Of the graduates who responded to the questionnaire, the majority came from Bahrain, representing 53 % (81 students) of respondents. The next highest proportions of students came from Canada (12 %) and the USA (5 %). Graduates represented a diverse array of nationalities, including Venezuela, Sri Lanka, Netherlands, Ghana, Germany, Brazil, and more.

From a satisfaction point view the mean rate of satisfaction ranged from 3.1 cross the item (Q11: The career guidance offered at RCSI Bahrain helped me gain the right type of experiences for my chosen career path) to 4.33 cross the item (Q32: RCSI Bahrain helped me to develop my skills to communicate effectively with patients and staff. Most of the results showed strongly agree with the level of readiness and satisfaction.

The highest rated aspect of the program was in response to the statement “I can establish and maintain constructive working relationships with colleagues” This item received a score of 4.42 out of a total of 5 (standard deviation ± 0.67). The lowest scoring item was “The career guidance offered at RCSI Bahrain helped me gain the right types of experiences for my chosen career path,” which scored a 3.10 (standard deviation ± 1.41).

Reliability analysis found high internal consistency for the total scale over the whole instrument items with a (Cronbach’s α of 0.97). The reliability analysis across the five factors was measured and it showed a high reliability (Cronbach’s coefficients 0.94, 0.92, 0.92, 0.80, 0.82) across the main five domains: future performance, career development, skills development, graduates as collaborator and communication skills respectively.

The whole instrument was found to be suitable for factor analysis (KMO = 0.853; Bartlett test significant, p < 0.00). Factor analysis showed that the data on the questionnaire decomposed into five factors, which accounted for 72.3 % of the total variance: future performance, career development, skills development, graduates as collaborator, and communication skills. The factors or scales established through exploratory factor analysis were used to establish the key domains (e.g., Future performance) for improvement, whereas the items within each factor provided more precise information about specific domain (e.g., My programme prepared me for lifelong learning, My programme helped me develop my leadership skills, etc.). This analysis made it possible to determine whether the instrument items were aligned into the appropriate constructs (factors) as intended (Table 2).

**Table 2:** Descriptive statistics for different scales

Scale	No of items	Mean	SD
Future performance	12	3.52	1.35
Career development	9	3.65	0.33
Skill development	10	4.05	0.22
Graduates as collaborator	7	4.24	0.17
Communication skill	3	4.08	0.20

Data were expressed as mean and SD

## Discussion

This study served to examine the satisfaction of RCSI Bahrain medical graduates, both with the program itself and with their own ability to translate what they had learned into application in real world practice.

### Feasibility

Despite low response rates, participants responded to nearly all items on the questionnaire. There were no questions that exceeded 20 % of the response “unable to assess” by the respondents, which indicates the feasibility of the survey. The data collected in this study allowed us to effectively and objectively evaluate the medical program offered by RCSI Bahrain. Medical student graduates are unique in that they are considered to be both novices and experts—novices in the sense that they are new to the clinical professional working world, yet experts in studying and learning relative to other students in non-medicine fields (Garner et al. 2014). This makes their feedback exceptionally valuable as their opinions should and will shape the curriculum for future students. The practice of using student feedback to improve on educational programs is one that dates back for nearly 50 years (Berk 2009). It has been found that student feedback is traditionally the primary source of evaluating faculty performance in universities across the United States (Brinkman et al. 2015).

### Psychometrics

It is important however, to consider that past studies have found that the self-reported confidence of medical students does not necessarily translate into higher competence in certain skills. A study conducted in Amsterdam found that students’ self-reported confidence in transcribing was weakly correlated with their actual competence in this key medical skill (Brinkman et al. 2015). This suggests that medical students may have difficulty in accurately self-evaluating their own skills and competencies (Lai and Teng 2011). As such, evaluations such as the medical graduate questionnaire should be taken in tandem with multisource evaluations that include assessment from sources such as fellow faculty members. (Berk 2009) suggests that feedback of medical programs should consider several evaluations including student, peer, outside experts, alumni, and administrators (Berk 2005).

### Learner satisfaction

This study found that student graduates from RCSI felt a strong sense of both satisfaction with their experience in the medical school, as well as a feeling of readiness to enter in the working world upon graduating. Although the word satisfaction was not used in the survey, it is used as a conclusion for the domains that were measured by the survey. If the graduates felt post-graduation that he/she had developed in different aspects such as future performance, career development, skills development and he/she ready as communicator and collaborator, this will subsequently lead to increased satisfaction levels since graduates master all the competencies required as students at the university. These findings may be limited due to the relatively small sample size due to low response rate, indicating possible respondent bias.

The information produced in this study will be fed back to university administrators such that the program may be adjusted as per student responses. Aspects of the program that resulted in low responses will be reviewed for their improvements for future cohorts and those areas that allowed for a strong satisfaction rate will continue to be monitored and emphasized throughout the curriculum.

## Limitations

This study had several limitations due to the relative newness of the RCSI Bahrain program. As such, only 153 students (a response rate of 26 %) completed the survey, limiting our sample size. The distribution of the questionnaire was done via university email. This may contribute to the low response rates, especially since students might not use their university e-mail on a regular basis after graduation. Additionally, a low response rate with disproportionate responses from more recent graduating classes suggests difficulty in establishing contact with alumni who graduate in previous years, as many may have changed their contact information from the time of graduation to the time of survey. Such a trend suggests a possible respondent bias, such that recent graduates are more likely to participate in such surveys. It is also possible that individuals who have higher satisfaction levels are more willing to participate in such research studies. This suggests limitations with the representativeness of the sample used in this study.

Secondly, while a student-centered evaluation method is an essential part of revising a medical education program, future studies might benefit from employing a 360° multisource feedback instead.
